# Combination Effects of Aminolevulinic Acid and Mycophenolic Acid on Hacat Cell Proliferation and Inhibition of Inosine Monophosphate Dehydrogenase

**DOI:** 10.3390/molecules30061359

**Published:** 2025-03-18

**Authors:** Manisha Venkatesh, Noelle Capriglione, Kaitlyn Rehberg, Jeffrey Voigt, Martha A. Hass

**Affiliations:** Department of Pharmaceutical Sciences, Albany College of Pharmacy and Health Sciences, 106 New Scotland Avenue, Albany, NY 12208, USA

**Keywords:** HaCaT cells, protoporphyrin IX, 5-aminolevulinic acid, photodynamic therapy, mycophenolic acid, IMPDH2 inhibition

## Abstract

Derivatives of mycophenolic acid (MPA) and 5-aminolevulinic acid photodynamic therapy (ALA-PDT) have been used separately to treat psoriasis, a chronic, inflammatory skin disease that is characterized by the unregulated hyperproliferation of epidermal keratinocytes and a T-cell-mediated immune response. However, the combination of these two therapies has not previously been explored. This study investigated the in vitro effects of combining MPA with ALA-PDT to suppress keratinocytes and the in vitro inhibition of inosine monophosphate dehydrogenase, a key enzyme. The effects of ALA, MPA, and their combination on protoporphyrin IX (PpIX) generation and cell viability in HaCaT cells, as well as the inhibition of IMPDH, were evaluated. Treatment of HaCaT cells with ALA, MPA, and their 1:1 molar combination showed that ALA alone induced PpIX production, with concentrations increasing from 5.25 ng/mL at 10 μM to 157.5 ng/mL at 1 mM. MPA did not increase PpIX on its own but had a modest synergistic effect with ALA at low concentrations (10 μM and 50 μM). The impact of blue light irradiation (465 nm) on cell viability was also assessed, revealing that ALA and ALA + MPA treatment led to significant reductions in HaCaT cell viability at higher concentrations (500 μM–1 mM), while MPA alone with blue light irradiation showed no cytotoxicity. The reduction in skin cell viability was enhanced when ALA was combined with MPA. Additionally, MPA effectively inhibited IMPDH activity in a dose-dependent manner, with 94–96% inhibition at concentrations of 100 μM and above. Interestingly, ALA weakly inhibited IMPDH, with a peak inhibition of 46% at 5 μM. At higher ALA concentrations, its inhibitory effect diminished, and it interfered with the potency of MPA’s IMPDH2 inhibition, suggesting that ALA could modulate MPA’s therapeutic action. These findings suggest that the combination of MPA with ALA-PDT may be a viable new treatment for psoriasis.

## 1. Introduction

Psoriasis is a non-infectious, chronic proinflammatory skin disorder that is characterized by relapsing and remitting episodes of excessively proliferating keratinocytes, scaly plaques, severe inflammation, and erythema [1,2]. The prevalence of psoriasis worldwide is 0.51% to 11.43% in adults and 0% to 1.37% in children [3]. The National Psoriasis Foundation has characterized mild psoriasis as lesions covering less than 2% of the body, moderate psoriasis as covering 2% to 10%, and severe psoriasis as covering more than 10% [4,5].

In recent years, strong evidence has emerged to suggest that the pathophysiology of psoriasis involves a sophisticated interplay between epidermal keratinocytes and both innate and adaptive immune responses [6]. The initial formation of psoriatic plaques is thought to involve the activation of recruited or resident T-cells that work in concert with dendritic cells in the skin to trigger inflammation, leading to altered differentiation and unregulated proliferation of keratinocytes [7,8]. Microvascular alterations are also apparent in psoriatic skin, characterized by excessive angiogenesis and distorted and dilated dermal blood vessels [9,10]. Keratinocytes secrete chemokines and growth factors that contribute to sustaining the inflammatory cascade by recruiting additional immune cells to the skin. Psoriasis may also be exacerbated by neuropeptides and pro-angiogenic factors that contribute to the inflammatory cascade and the excessive proliferation of keratinocytes, enhancing angiogenesis and vasodilation [11,12]. Thus, therapies for treating psoriasis are aimed at suppressing the hyperproliferation of keratinocytes, tempering the activation and infiltration of immune cells in the skin, and limiting the secretion and action of signaling molecules believed to be associated with the progression of the disease [13].

Treatment modalities involve single agents or combination therapies delivered topically to the skin or administered systemically [14,15,16]. Topical agents, including corticosteroids (betamethasone and fluticasone), vitamin D analogs, and phototherapy, are effective for the treatment of mild psoriasis [17]. Treatment of moderate or severe psoriasis generally requires systemic agents such as methotrexate (MTX), biologics, or immune suppressants such as mycophenolic acid (MPA) derivatives [18]. Among the most effective systemic therapies for treating severe or refractory psoriasis are biologics that act as immune antagonists [19,20]. The overall safety and efficacy of these agents have generally been good; however, serious side effects, notably the risk of infections, have been encountered with some of these agents [21]. Additionally, the high cost and the necessity of administering biologics parenterally are viewed as limitations to their use [22].

Mycophenolic acid (MPA) was originally used to treat psoriasis in the 1970s, but severe gastrointestinal side effects halted its use until a prodrug of MPA, mycophenolate motefil (MMF), was developed in 1995. MMF, a 2-morpholinoester of MPA, undergoes metabolic hydrolysis to MPA upon oral or parenteral administration and has improved bioavailability and significantly fewer side effects than MPA itself [23,24]. MPA exhibits efficacy in psoriasis through preferential inhibition of the type II isoform of inosine monophosphate dehydrogenase (IMPDH), a key enzyme in the synthesis of purine. The type II isoform of IMPDH is highly expressed on T-cells; thus, its inhibition deprives the T-cells of purine and guanosine nucleotides needed for the synthesis of DNA, RNA, and proteins, resulting in an anti-inflammatory effect [25,26].

**Figure 1:**
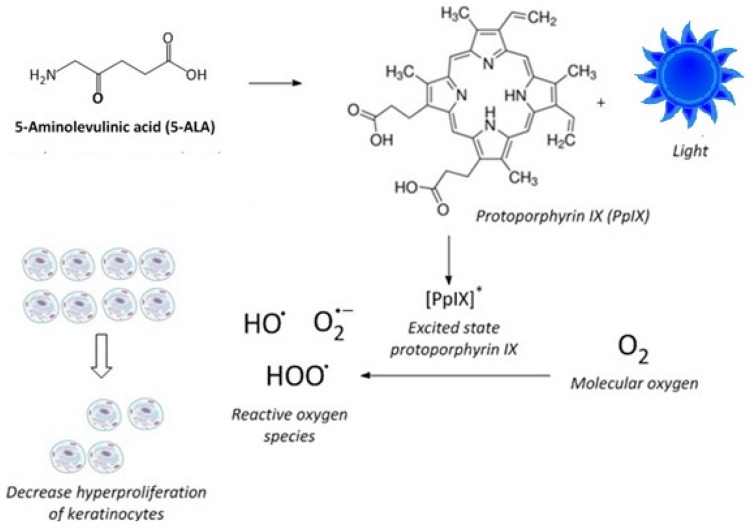
Mechanism of photodynamic therapy using ALA. ALA results in increased PpIX, which, when irradiated, produces PpIX in the excited state, which generates reactive oxygen species (ROS). These ROS limit the proliferation of keratinocytes.

In addition to chemotherapeutic agents, phototherapy has long been used to treat psoriasis, either alone or in combination with other systemic or topical treatments. Specifically, photodynamic therapy (ALA-PDT) using 5-aminolevulinic acid (ALA) as a photosensitizing precursor, coupled with exposure to blue light, modulates the hyperproliferation of keratinocytes [27,28,29]. A is a prodrug that, when administered to mammalian cells or tissues, increases the concentration of protoporphyrin IX (PpIX) [30,31,32]. PpIX is a precursor to heme and is an endogenous photosensitizer [33]. Blue light irradiation of cells or skin tissue, pretreated with ALA to elevate PpIX levels, promotes the formation of PpIX in the excited state, which, upon reaction with molecular oxygen, forms cytotoxic reactive oxygen species that modulate the hyperproliferation of keratinocytes [34] (Figure 1). Combining phototherapy with topical and systemic drugs is frequently used in the treatment of psoriasis and some cancers to optimize efficacy and minimize toxicity by lowering the effective doses [14]. However, the combination of MPA or its derivatives with ALA-PDT has not been previously investigated.

Consistent with strategies to optimize treatment outcomes using combination therapy and limit systemic side effects for psoriasis patients [14,15,16], we explored how the combination of ALA-PDT with MPA affects the proliferation of keratinocytes and the in vitro inhibition of IMPDH. Using immortalized human keratinocytes (HaCaT cells), we assessed the impact of MPA treatment combined with ALA-PDT on the generation of PpIX and subsequent cell viability after treatment. The effect of IMPDH inhibition by MPA in combination with ALA was also evaluated using a human inosine-5′-monophosphate dehydrogenase (IMPDH) inhibitor screening assay kit. Through these studies, we aim to determine if the combination of MPA with ALA-PDT is a viable treatment to simultaneously suppress the hyperproliferation of keratinocytes and the cell-mediated immune response. Our longer-term aim is to develop novel combination prodrugs (or co-drugs) that incorporate ALA and MPA in a 1:1 molar ratio; thus, we used this molar ratio in these studies.

## 2. Results

### 2.1. Calibration Curve for PpIX

**Figure 2:**
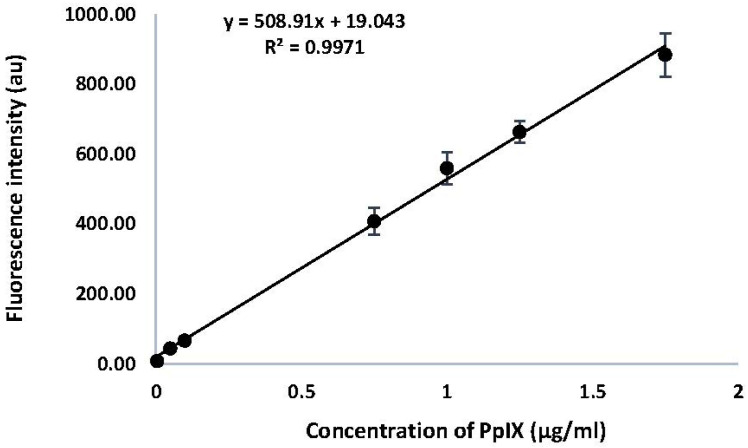
Calibration Curve for PpIX. Fluorescence intensity at λ_ex_ 400 nm was monitored from 500 nm–700 nm and recorded at λ_em_ 605 nm. Results are shown as a mean (*n* = 3) and reported as ±SEM, (*p* < 0.05).

A calibration curve was generated for PpIX by measuring the fluorescence intensity of emission at 605 nm (λ_ex_ 400 nm). Results are shown in Figure 2. A linear relationship was observed between the fluorescence intensity of emission for PpIX, with a correlation co-efficient of 0.9971 across the range of concentrations measured. The equation derived from this calibration curve was used to determine the concentrations of PpIX in HaCaT cells in response to drug treatments with ALA and MPA.

### 2.2. HaCaT Cells Treated with ALA, MPA, and ALA + MPA

The MTT assay was carried out to assess the viability of cells in response to treatment with ALA, MPA, and a 1:1 molar ratio of ALA + MPA in concentrations up to 1 mM. DMSO (1% in PBS) was used as the vehicle and control, and de-ionized (DI) water was used as the blank, representing fully viable cells. Samples were run in triplicate. No loss of cell viability was observed in the DMSO in the PBS control relative to the blank. Cell viability was maintained in all samples treated with ALA alone, MPA alone, and 1:1 molar concentrations of ALA + MPA at all concentrations evaluated up to 1 mM over a 4 h time course. Samples were run in triplicate.

PpIX was measured in HaCaT cells treated with ALA, MPA, and ALA + MPA in concentrations ranging from 1 μM–1000 μM, using 1% DMSO in PBS as the vehicle. PpIX was not detected in HaCaT cells treated with the vehicle alone. Similarly, no PpIX was detected in the HaCaT cells when they were treated with the two lowest concentrations of ALA (1.0 µM and 5.0 µM). However, when HaCaT cells were treated with 10 µM of ALA, 5.25 ng/mL of PpIX was detected, with a steady rise in PpIX generation as ALA concentrations were increased. The amount of PpIX generated in HaCaT cells treated with 50 µM, 100 µM, 500 µM, and 1000 μM of ALA was 34.1, 66.69, 103.7, and 157.5 ng/mL, respectively.

**Figure 3:**
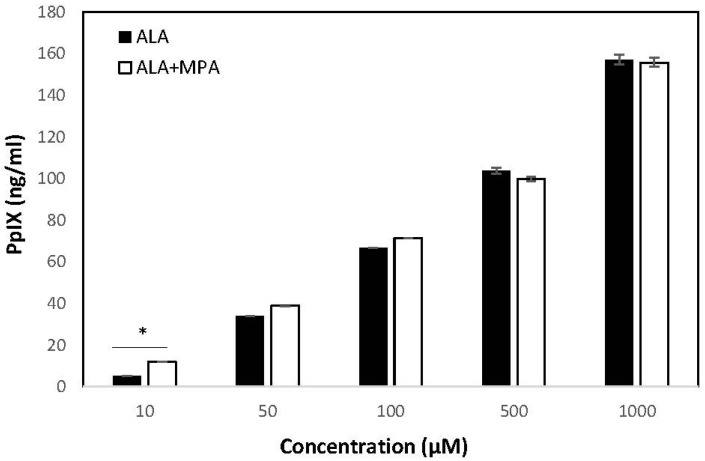
Concentrations of PpIX in HaCaT cells in response to treatment with ALA and ALA + MPA. Concentrations of PpIX generated in HaCaT cells (600 K) in response to treatment with increasing concentrations of ALA and ALA + MPA with 1% DMSO/PBS as the solvent (1.0 µM–1 mM). PpIX concentrations were measured using a fluorescence spectrophotometer (λ_ex_ 400 nm, λ_em_ 605 nm). Results are shown as a mean and ± SEM, * *p* < 0.05.

PpIX concentrations in HaCaT cells were measured using the same vehicle, cell count, and time conditions after treatment with MPA and 1:1 molar combinations of ALA + MPA across the same range of concentrations reported for ALA alone. As expected, MPA did not induce the production of PpIX in HaCaT cells at all concentrations evaluated. At the lowest concentrations of ALA + MPA (1.0–5.0 µM), no PpIX was detected, consistent with what was observed with ALA alone. However, treatment of HaCaT cells with 10 µM and 50 µM ALA resulted in concentrations of PpIX of 11.98 ng/mL and 38.76 ng/mL, respectively. At 100 µM and 500 µM, PpIX concentrations in HaCaT cells were 71.29 ng/mL and 99.83 ng/mL, respectively. At the highest concentration of ALA + MPA (1 mM), the amount of PpIX measured was 155.77 ng/mL. Concentrations of PpIX in response to treatment with ALA alone and the combination of ALA and MPA across the range of treatment concentrations are shown in Figure 3.

The amount of PpIX generated by treatment with ALA alone, MPA alone, and equivalent concentrations of 1:1 molar combinations of ALA + MPA were compared to determine if the combination of ALA with MPA was synergistic, null, or antagonistic. The levels of PpIX in cells simultaneously treated with the combination of ALA + MPA were compared with the sum of PpIX concentrations found with ALA alone plus those found with MPA alone across concentrations ranging from 1 µM–1 mM. Cells treated with concentrations of 10 µM and 50 µM of ALA alone generated 5.25 ng/mL and 34.08 ng/mL of PpIX, respectively. Treatment of cells with ALA combined with MPA (10 µM) produced 11.98 ng/mL of PpIX, which was significantly more than ALA alone. Approximately the same amount of PpIX was detected in cells treated with the combination of ALA and MPA at concentrations of 50 µM, 100 µM, and 1 mM. These data suggest that MPA does not inhibit the ALA-induced generation of PpIX in HaCaT cells (i.e., has a null effect) and it may have a modest synergistic effect at concentrations of 10 µM when used in a 1:1 molar ratio with ALA. Data comparisons are shown in Table 1.

### 2.3. Effect of Blue Light Irradiation on the Viability of HaCaT Cells Treated with ALA, MPA, and ALA + MPA

The viability of HaCaT cells treated with ALA, MPA, and ALA + MPA across a range of concentrations (250, 500, and 1000 μM) with and without blue light irradiation was determined using the MTT assay. Cells treated with the vehicle or de-ionized water and exposed to blue light alone (465 nm LED 14 W/cm^2^) for 20 min did not show significantly reduced cell viability compared with non-irradiated cells. This irradiation dose was used in experiments on HaCaT cells treated with ALA alone, MPA alone, or ALA + MPA. The viability of HaCaT cells treated with 250 μM ALA followed by exposure to blue light for 20 min was not significantly reduced relative to the control. However, the viability of HaCaT cells treated with 500 μM or 1 mM ALA and exposed to blue light for 20 min was significantly lower than treated cells with no irradiation. When HaCaT cells were treated with MPA alone at the same concentrations and exposed to blue light at the same dose, HaCaT cell viability was not significantly reduced. Pretreatment with ALA + MPA in concentrations of 250 μM, 500 μM, and 1 mM combined with blue light irradiation resulted in significant decreases in cell viability relative to non-irradiated cells (Table 2). However, the combination of ALA-PDT with MPA did not significantly reduce cell viability relative to ALA-PDT alone.

### 2.4. Inhibition of IMPDH with MPA, ALA, and MPA + ALA

The in vitro inhibition of IMPDH with MPA, ALA, and the combination of MPA + ALA (1:1 molar concentration) in 1% DMSO/PBS was measured using a colorimetric kit (Table 3). DMSO (1% in PBS) was used as the vehicle and control and samples were run in triplicate. The vehicle did not affect enzyme activity. The doses used in these experiments are at the higher end of the concentration range recommended by the manufacturer. We used these doses to match the dose concentrations used in the experiments to measure PpIX in HaCaT cells.

The inhibition of IMPDH by MPA at concentrations ranging from 1.0–1000 µM was determined. MPA concentrations of 1 µM, 5 µM, 10 µM, and 50 µM resulted in 79%, 84%, 85%, and 94% inhibition of IMPDH, respectively. At concentrations of 100 μM and 500 μM MPA, inhibition of IMPDH was 95% and 96%, respectively. The highest concentration of MPA (1000 µM) showed an inhibition of 96%.

Inhibition experiments were then run under the same conditions with ALA and 1:1 molar combinations of ALA + MPA across the same range of concentrations reported for MPA alone. At the lowest concentration of ALA (1.0 µM), IMPDH was inhibited by 35%. As the concentration of ALA was increased to 5 µM, IMPDH inhibition increased to 46% and remained constant at 46%, 40%, and 37% with ALA concentrations of 10 µM, 50 µM, 100 µM, and 500 µM, respectively. At the highest concentration of ALA (1000 µM), no inhibition of IMPDH was observed. ALA was not expected to inhibit IMPDH at any concentration. However, these results suggest that ALA moderately inhibits IMPDH (i.e., ~40%) at concentrations ranging from 1–500 µM.

A dose–response curve for the inhibition of the IMPDH enzyme by 1:1 MPA + ALA was generated using concentrations ranging from 1.0–1000 µM. At the lowest concentrations of MPA + ALA (1.0 and 5.0 µM), the inhibition observed was 91% and 90%, respectively. At concentrations of 10 µM and 50 µM, the IMPDH inhibition observed was 92% and 92%, respectively, approximately the same as the inhibition observed at lower concentrations. At a concentration of ALA + MPA of 100 µM, the IMPDH inhibition attained was 83%. At a concentration of 500 µM, the inhibition dropped to 62%, and at the highest concentration of 1000 µM, there was a significant drop in inhibition to 7%.

The inhibition of IMPDH by MPA alone and equivalent concentrations of a 1:1 molar combination of MPA + ALA were compared to determine if the combination of ALA with MPA was synergistic, null, or antagonistic. At low concentrations (1, 5, and 10 µM), a statistically significant difference in IMPDH inhibition by MPA + ALA compared to MPA alone was observed, suggesting that ALA may enhance IMPDH inhibition relative to MPA alone at these lower concentrations. At a concentration of 50 µM, IMPDH inhibition with MPA alone was 94%, and for MPA + ALA, it was 93%, which is not statistically different. IMPDH inhibition was 96% at concentrations of both 100 µM and 500 µM of MPA alone, and 84% and 62% for MPA + ALA, respectively. At these higher concentrations, the presence of ALA significantly reduced IMPDH inhibition relative to that observed with MPA alone. Similarly, at the highest concentration of 1000 µM, inhibition of IMPDH with MPA alone was the highest at 96%, whereas it dropped significantly to 7% for MPA + ALA, suggesting that ALA interferes with the ability of MPA to inhibit IMPDH at this concentration.

## 3. Discussion

HaCaT cells were used to assess the effect of ALA-PDT, both alone and in combination with MPA, on the generation of PpIX and cell proliferation. HaCaT cells are a useful model to investigate anti-inflammatory interventions/therapies for skin diseases such as psoriasis. Wang et al. demonstrated that treatment of HaCaT cells with ALA-PDT showed pro-apoptotic effects by enhancing cell apoptosis and upregulating the apoptotic genes PARP and caspase 3 [35]. Others have used this cell line to correlate ALA dosage with PpIX concentrations, showing a positive correlation between increased PpIX levels and inhibition of cell proliferation [27,36]. Consistent with these prior studies, we used this cell line as a suitable model to study the combined effects of ALA and MPA in vitro [37].

To ensure that the drug treatments were not cytotoxic to HaCaT cells, MTT assays were run on HaCaT cells treated with ALA, MPA, and a 1:1 molar combination of both drugs, with concentrations up to 1 mM, for 4 h. The MTT assay is a colorimetric assay to assess cellular metabolic activity and is frequently used to assess cell viability and drug cytotoxicity [38]. In addition to using this assay to assess the cytotoxicity of ALA and MPA on HaCaT cells, we used the MTT assay to determine the impact of blue light exposure on cell viability with and without treatment with ALA, MPA, and ALA [39]. Results from this assay showed that ALA and MPA are not cytotoxic to HaCaT cells and hence could be used in experiments to evaluate the effect of MPA on the ALA-induced generation of PpIX and cell viability after drug treatment and blue light irradiation.

Concentrations of PpIX in HaCaT cells treated with ALA, MPA, and a 1:1 molar combination of ALA and MPA were measured using fluorescence spectroscopy to determine the effect of MPA on the ALA-induced generation of PpIX in cells. We found that ALA increased the concentration of PpIX in HaCaT cells in a dose-dependent manner, consistent with previous reports [27]. ALA is a prodrug that increases the concentration of the endogenous photosensitizer, PpIX, in skin cells [40] and skin tissue [30]. Results from our experiments involving the treatment of HaCaT cells with ALA alone, MPA alone, and ALA + MPA revealed that MPA does not induce the generation of PpIX in HaCaT cells across the range of concentrations evaluated, nor does MPA cause a reduction in the generation of PpIX induced by ALA. A surprising finding was that at low concentrations (10 μM), MPA appears to have a synergistic effect with ALA to elevate PpIX levels above those predicted by an additive effect alone. PpIX levels were significantly higher with ALA +MPA than with ALA at a concentration of 10 μM. At higher concentrations, the combination effect was not significant, but MPA did not interfere with the ALA-induced elevation of PpIX. These data suggest that MPA could be used effectively in combination with ALA-PDT to treat moderate to severe psoriasis.

Once we established that ALA and the combination of ALA with MPA elevates PpIX in HaCaT cells, we explored whether blue light irradiation of treated cells would result in reduced cell viability. In our experiments with ALA-treated HaCaT cells, irradiation with blue light reduced cell viability at concentrations of 500 μM and 1 mM. This finding is consistent with studies performed by Ge et al., who also found that when HaCaT cells were treated with ALA concentrations of 2 mM, cell viability decreased after irradiation [27]. MPA alone did not affect cell viability even with blue light irradiation. The reduction in cell viability induced by ALA and blue light irradiation was maintained but not enhanced in the presence of MPA relative to ALA treatment alone at concentrations of 250 μM, 500 μM, and 1 mM. These results align with the observed increase in PpIX with ALA + MPA treatment and further support the viability of developing combination therapies with these two drugs.

We then explored whether ALA impacted the inhibitory activity of MPA on IMPDH. MPA exhibits efficacy in psoriasis through preferential inhibition of the type II isoform of inosine monophosphate dehydrogenase (IMPDH2), a key enzyme in the synthesis of purines [24,25]. IMPDH2 is highly expressed in activated T-cells, and the selective inhibition of IMPDH2 by MPA results in a more potent cytostatic effect on activated T-cells than other cells in the body [41]. Using an IMPDH inhibitor screening assay kit, we evaluated the inhibitory activity of MPA alone, ALA alone, and MPA + ALA in a 1:1 molar ratio. As expected, MPA inhibited IMPDH, which is consistent with previous reports and the guidelines published by the manufacturer [25]. To our surprise, ALA also weakly inhibited IMPDH at concentrations ranging from 1–500 μM (34–46% inhibition). At low concentrations (1–10 μM), ALA significantly increased the inhibition of IMPDH relative to MPA alone. However, at higher concentrations, ALA appears to interfere with the inhibitory activity of MPA. These findings suggest that combining ALA-PDT with MPA at low doses may enhance the therapeutic benefit of MPA for treating psoriasis.

## 4. Materials and Methods

HaCaT cells used in the experiments were purchased from Addex Bio (San Diego, CA, USA). The cell culture flasks used to culture the adherent HaCaT cells were purchased from Corning (Tewksbury, MA, USA). The cell culture plates used for the experiments were purchased from Celltreat (Pepperell, MA, USA). Chemical reagents, including 5-aminolevulinic acid (ALA), mycophenolic acid (MPA), protoporphyrin IX (PpIX), Dulbecco’s modified Eagle medium (DMEM), trypsin, fetal bovine serum (FBS), MTT reagent, the solubilizing agent used for MTT assays, and perchloric acid, were purchased from Sigma-Aldrich (St. Louis, MO, USA). Penicillin–streptomycin was purchased from ThermoFisher Scientific. (Waltham, MA, USA). The FBS added into DMEM was purchased from Corning (Tewksbury, MA, USA). The human inosine-5′-monophosphate dehydrogenase (IMPDH) inhibitor screening assay kit was purchased from Biovision (Milpitas, CA, USA). DMSO used as a solvent was purchased from Sigma Aldrich. An inverted Olympus CK2 microscope (Olympus-Life Science Solutions, Waltham, MA, USA) was used to observe the cells for confluency in T-flasks and 6-well plates. A Hermle Z360 centrifuge, a Hermle Labor Technik and Eppendorf 5415D centrifuge (Hauppauge, NY, USA), and a Reichert-Bright Line^®^ hemacytometer (Buffalo, NY, USA) were used to process cells. The fluorescence of PpIX, the optical density (OD) for the IMPDH assay, and absorbances for the MTT assays were measured using a Synergy 2 Biotek plate reader (Winooski, VT, USA). A 465 nm LED (blue light, 14 W/cm^2^) light source used for different durations was used for irradiation experiments.

### 4.1. Culturing HaCaT Cells

To maintain a supply of cells, the cells were stored frozen in liquid nitrogen and thawed as necessary to complete experiments. All experiments were carried out in a biosafety hood to maintain sterility and avoid cross-contamination. Once thawed, the cells (1 mL) were transferred to a T-25 flask containing 10% FBS supplemented DMEM (complete media, 7 mL), 1% penicillin (10,000 U/mL), and streptomycin (10,000 μg/mL) and incubated at 37 °C with 5% CO_2_. Confluent HaCaT cells were passaged every 2–3 days by first adding trypsin (3 mL) to release attached cells from the sides of the T-25 flask. Once all cells were detached, the culture was transferred to a centrifugation tube and centrifuged at 900 rpm for 5 min to create a cell pellet at room temperature (25 °C). After carefully discarding the supernatant, the cell pellet was resuspended into fresh complete media (3 mL) using a sterile pipette. A few drops of the resuspended culture were transferred to a new, sterile T-25 flask. The culture was titrated with 10% FBS DMEM media (up to 8 mL) and stored at 37 °C. To determine the cell concentration, an aliquot (50 μL) of the previously resuspended culture was carefully transferred to a hemacytometer to count cells.

### 4.2. Cryopreservation of HaCaT Cells

When the cells were ready to be passaged for the fourth time, cryopreservation took place. The spent media was removed and trypsin EDTA was added. The cells were incubated for 6 min until the cells were unadhered from the flask. Complete media was added to the flask to neutralize the trypsin. Using a hemocytometer, the cells were counted and then centrifuged for 5 min at 900 rpm. The supernatant was removed, and the cells were resuspended in freezing media (10% DMSO + 90% DMEM, prepared in the lab) to make the cell density in each cryovial 1.5 × 10^6^ cells per milliliter. The cell suspension (1 mL) was added to cryovials, resulting in the production of 44 cryovials, each containing 1.5 × 10^6^ cells. The cryovials were placed in a controlled-rate freezing container in a −80 °C freezer to ensure that freezing occurred at −1 °C per minute. After 24 h, the cryovials were transferred and stored in liquid nitrogen.

### 4.3. MTT Assay: Cell Viability

The MTT assay was used to assess the cell viability and drug cytotoxicity of ALA and MPA on HaCaT cells by measuring cellular metabolic activity. Resuspended cells (1 mL) were added to the hemocytometer and cells were counted. The average number of cells per ml was calculated and dilutions were made as required for each experiment (~30,000 cells/mL for the MTT assay). Once the dilutions were made, the cell solution (~30,000 cells/mL) was added to the wells in a 48-well plate and incubated overnight. After 24 h, the medium was removed and replaced by drug solutions of ALA or MPA (10 µL, 0.001 mM–1 mM) and a 1:1 molar ratio of ALA and MPA (5 µL each, 0.001 mM–1 mM). Serum-free media was added to each well to reach a total volume of 200 mL. After incubating for 2 h, 10% of the total volume (20 µL) was removed and replaced with MTT (0.5 g/mL, 20 µL) in each well.

The cells were incubated for another 2 h following the addition of MTT. Media was removed, and solubilizing buffer (200 µL) was added. Cell viability was measured using a plate reader. Absorbance was measured at 570 nm and 690 nm. The optical density (OD) was measured at 570 nm and 690 nm (reference wavelength). Cells were plated in triplicate for each concentration of the drug. The % cell viability was calculated using the formula (Equation (1)):

(1) $$\%\;cell\;viability=\left[\left({abs}_{sample}-{abs}_{blank}\right)/\left({abs}_{control}-{abs}_{blank}\right)\right]\times 100$$

### 4.4. Calibration Curve of PpIX

A stock solution of commercial PpIX (0.89 mM) was prepared in 1 M HClO_4_ (50:50 *v*/*v* methanol/water). The stock solution was serially diluted with the same solvent to prepare samples (20–200 nM) for analysis using fluorescence spectroscopy. Samples were covered with aluminum foil to avoid light exposure. Fluorescence intensity at 605 nm for each sample concentration was measured using an excitation wavelength of 400 nm. Each sample concentration was run in triplicate. The PpIX concentration was plotted against the fluorescence intensity to generate the calibration curve. The curve was used to quantify the amount of PpIX produced by cells treated with ALA, MPA, and 1:1 molar concentrations of ALA + MPA.

### 4.5. Measurement of PpIX in HaCaT Cells

Cells were treated with ALA, MPA, or a combination of ALA + MPA (1: 1 molar concentration). The concentrations of ALA sufficient to generate a robust and reproducible fluorescence intensity of PpIX were established and then the same molar concentrations were used for MPA and the combination of ALA + MPA. Cells (600,000 in 2 mL of media) were plated in each well of a 6-well plate and incubated overnight at 37 °C to allow cells to reach confluency. The media was then removed and replaced with serum-free media containing ALA, MPA, or ALA + MPA (200 µL) of varying concentrations (1 µM–1 mM). Serum-free media (1.8 mL) was then added to each well to bring the total volume to 2 mL. Plates were covered with aluminum foil and incubated for 4 h at 37 °C. After 4 h, the plates were brought to a dark room, the serum-free media was removed, and each well was washed twice with PBS (2 × 1 mL). HClO_4_ (1 mL, 1 M in 50% methanol–water) was added to each well to solubilize the cells. Cells were scraped from the wells, transferred to microfuge tubes, and centrifuged for 5 min at 13000 rpm to pellet the cell debris. After centrifugation, the supernatant was transferred to a cuvette and the fluorescence intensity of each sample was quantitated using fluorescence spectroscopy (λ_ex_ 400 nm, λ_em_ 605 nm).

### 4.6. Cell Viability After Exposure to Blue Light

The viability of HaCaT cells was measured using the MTT assay after treatment with ALA alone, MPA alone, and the combination of ALA with MPA (1:1 molar ratio) with and without blue light exposure. Untreated HaCaT cells were irradiated with a 465 nm LED (blue light, 14 W/cm^2^) with cells positioned 9 cm from the source. An appropriate irradiation dose that did not reduce cell viability on its own was established.

ALA and MPA solutions were prepared in PBS as the vehicle for the drug treatment of the cells. Cells were plated in two separate 48-well plates (20,000 cells/well) in 10% FBS media (total volume 1 mL) and allowed to incubate for 1 h at 37 °C. Cells were treated with ALA, MPA, or ALA + MPA at concentrations of 250 μM, 500 μM, and 1000 μM and allowed to incubate for 4 h in the dark at 37 °C. Each drug concentration was run in triplicate along with a PBS control. After the 4 h incubation, the cell media was replaced with fresh complete media. One plate was irradiated with blue light (465 nm) for 20 min in a closed incubation chamber and the other plate remained covered in foil in a separate incubator. Following the blue light exposure, the cells were allowed to incubate for 1 h in the dark at 37 °C and the media was replaced with fresh media containing a 10% MTT reagent. Following incubation for an additional hour, the cells were processed for the quantitation of formazan, as described above.

### 4.7. Inhibition of IMPDH

The inhibition kit was stored at −20 °C. The contents of the kit were thawed prior to use and the enzyme was kept in ice at all times. IMPDH was reconstituted with 200 µL of assay buffer and the IMPDH substrate was reconstituted with 1.2 mL of assay buffer. Serial dilutions of MPA, ALA, or MPA + ALA (1:1 molar ratio) concentrations ranging from 0.001 mM–1 mM were prepared in a 20% DMSO assay buffer, such that the final concentration of DMSO in each well was 1%. Each concentration of MPA, the solvent control (20% DMSO assay buffer), and blanks (10 µL each) were run in triplicate. The enzyme solution mix (178 µL) was added to each well containing 10 µL of the candidate solutions. The candidate compounds were incubated with the enzyme solution for 30 min at room temperature. After 30 min, the substrate mix (12 µL) was added to each well. The final reaction volume in each well was 200 µL. The optical density (OD) was measured at 450 nm at 37 °C for 60 min in kinetic mode, at intervals of 2 min and plotted (time versus OD450). The slope of the control and the slope of the samples were determined from the equation (y = mx + b, m = slope) of the linear curve generated from the plot of OD450 versus time. 

Inhibition (%) was calculated using the formula [(slope of control − slope of sample)/slope of control] × 100. Relative activity (%) was calculated using the formula (slope of sample/slope of control) × 100. The dose–response relationship between the IMPDH enzyme and MPA concentrations was analyzed and plotted.

## 5. Conclusions

The results presented demonstrated that at concentrations of 10 µM, a 1:1 molar combination of ALA and MPA increased PpIX levels (compared to ALA alone) in HaCaT cells. At higher concentrations, the combination of MPA with ALA did not interfere with the observed ALA-induced increase in PpIX levels in HaCaT cells. Results from the cell viability studies with ALA-PDT or ALA + MPA and blue light irradiation suggested that the levels of PpIX produced were sufficient to reduce cell viability. While MPA did not significantly affect the reduction in cell viability induced by ALA-PDT, it also did not interfere with the ability of ALA-PDT to suppress cell proliferation. Inhibition of IMPDH with a 1:1 molar combination of MPA + ALA (compared to MPA alone) was enhanced at lower concentrations (1–10 μM). However, at higher concentrations, ALA interfered with MPA’s inhibition of IMPDH. These data suggest that combining ALA with MPA at low concentrations may provide enhanced efficacy in limiting keratinocyte proliferation through photodynamic therapy and modulating the immune response through inhibition of IMPDH and limiting T-cell replication. While not explored in this study, these therapies may also impact the cascade signaling molecules that contribute to inflammation and the microvascular distortions associated with psoriasis.

## Figures and Tables

**Table 1:** Amount of PpIX generated by ALA, MPA, and ALA + MPA (observed and additive). Concentrations range from 1.0 µM–1 mM, with 1% DMSO/PBS as the solvent. Results are shown as a mean and ± SEM. ** *p*-value ≤ 0.05.

Concentration (µM)	PpIX Concentration (ng/mL)			
	ALA	MPA	ALA + MPA (OBS)	ALA + MPA (ADD)
1.0	ND *	ND	ND	
5.0	ND	ND	ND	
10	**5.26 ± 1.33**	ND	11.98 ± 1.14 **	**5.26 ± 1.33**
50	34.08 ± 2.30	ND	38.76 ± 2.17	34.08 ± 2.30
100	66.69 ± 1.02	ND	71.28 ± 3.30	66.69 ± 1.02
500	103.71 ± 12.96	ND	99.83 ± 4.46	103.71 ± 12.96
1000	157.15 ± 3.21	ND	155.77 ± 1.7	157.15 ± 3.21

Note: Bold formatting is used to emphasize key differences between groups. * ND = not detected.

**Table 2:** Viability of HaCaT cells treated with ALA, MPA, and ALA + MPA with and without blue light irradiation, where 1% DMSO/PBS was used as the vehicle and de-ionized water was used as the blank. Results are shown as a mean and ±SEM.

	% Cell Viability					
Concentration	250 μM		500 μM		1 mM	
465 nm Irradiation	**−**	**+**	**−**	**+**	**−**	**+**
1% DMSO/PBS	100% ± 3.0	100% ± 3.1	100% ± 2.2	92 ± 2.1%	100% ± 3.7	94% ± 4.4
MPA	90% ± 2.3	85% ± 3.3	91% ± 5.1	87% ± 5.1	88% ± 2.8	84% ± 6.1
ALA	93% ± 4.2	87% ± 4.9	100% ± 6.1	70% ± 4.4	100% ± 5.4	56% ± 7.1
ALA + MPA	90% ± 5.1	74% ± 4.3	90% ± 5.1	60% ± 7.1	86% ± 6.1	54% ± 5.8

**Table 3:** **Additive response effect of ALA and MPA on IMPDH inhibition.** Inhibition of IMPDH by MPA, ALA, and ALA + MPA (observed and additive), with concentration ranges from 1.0 µM–1000 µM in 1% DMSO/PBS. Results are shown as a mean and ±SEM. ** *p*-value ≤ 0.05.

Concentration (µM)	IMPDH Inhibitory Activity (%)			
	ALA	MPA	ALA + MPA (OBS)	ALA + MPA (ADD)
1	34.51 ± 5.68	**78.86 ± 0.33**	90.94 ± 10.8 **	113.38 ± 5.41
5	45.75 ± 12.5	**84.02 ± 0.11**	90.43 ± 2.13 **	129.77 ± 12.45
10	39.44 ± 8.3	**85.47 ± 0.53**	92.48 ± 2.23 **	124.92 ± 7.9
50	46.15 ± 18.62	93.61 ± 0.09	92.82 ± 0.51	139.76 ± 18.6
100	39.45 ± 4.74	**95.63 ± 0.87**	83.60 ± 1.06 **	135.08 ± 4.53
500	36.68 ± 4.62	**95.42 ± 0.67**	62.26 ± 2.07 **	132.11 ± 4.01
1000	−16.76 ± 1.48	**96.16 ± 0.75**	6.42 ± 2.07 **	79.40 ± 1.29

Note: Bold formatting is used to emphasize key differences between groups.

## Data Availability

The original contributions presented in this study are included in the article. Further inquiries can be directed to the corresponding author.

## Abbreviations

The following abbreviations are used in this manuscript:

MPA	Mycophenolic acid
ALA	5-Aminolevulinic acid
MFF	Mycophenolate motefil
IMPDH	Inosine monophosphate dehydrogenase
ALA-PDT	5-Aminolevulinic acid photodynamic therapy
PpIX	Protoporphyrin IX
DMSO	Dimethylsulfoxide
PBS	Phosphate-buffered saline
